# Supplementary material for: Machine Learning Detects Pattern of Differences in Functional Magnetic Resonance Imaging (fMRI) Data between Chronic Fatigue Syndrome (CFS) and Gulf War Illness (GWI)
Source: Brain Sci. 2020 Jul 17;10(7):456. doi: 10.3390/brainsci10070456 (PMC7407325; doi:10.3390/brainsci10070456)
Supplement: Supplementary file 1 [file brainsci-10-00456-s001.zip › brainsci-840068-supplementary.pdf]

Supplementary Material: Machine Learning Detects Pattern of Differences in Functional Magnetic Resonance Imaging (fMRI) Data between Chronic Fatigue Syndrome (CFS) and Gulf War Illness (GWI)

Destie Provenzano [1,2], Stuart D. Washington [1], Yuan J. Rao [3], Murray Loew [2] and James Baraniuk [1,*]

Table S1: Comparison of all regions from this analysis and our two prior analysis as referenced in Provenzano et al (2020)

Abbreviation	CFS vs GWI Day 1	CFS vs GWI Day 2	GWI vs SC Day 1	GWI vs SC Day 2	CFS vs SC Day 1	CFS vs SC Day 2
Frontal_Sup_Orb_R	-0.59	-0.1	0.0627	0.0245		-0.039
Frontal_Mid_Orb_L	-0.44		0.2989		0.193	
Cingulum_Mid_R	-0.28					
Occipital_Mid_R	-0.23		-0.0461		-0.262	
Frontal_Inf_Oper_L	-0.08					
SupraMarginal_L	-0.07		0.1442	0.0213	0.294	-0.008
Occipital_Mid_L	-0.07	-0.1				0.203
Precuneus_R	-0.05	0.04				0.107
Fusiform_R	-0.04				0.269	
Frontal_Inf_Tri_R	-0.02	-0.2				-0.148
Parietal_Sup_R	-0.02	-0.06				
Frontal_Mid_R	-0.02	-0.02				
Frontal_Inf_Orb_R	-0.02					
Parietal_Inf_L	-0.01	-0.06				
Precuneus_L	0.01	-0.13				-0.172
Temporal_Inf_R	0.03	0.07				
Parietal_Sup_L	0.04	-0.09				
Frontal_Mid_L	0.04	0.06				
Frontal_Sup_R	0.05	0.16			0.107	
Frontal_Sup_L	0.06		-0.0646		-0.117	
Angular_R	0.08	0.04	0.0399			
Insula_R	0.13	0.26	-0.0368	-0.0131		
Postcentral_R	0.13	0.21		-0.0247	-0.379	-0.161
Rolandic_Oper_R	0.14				0.534	

Cingulum_Mid_L	0.15			0.0945		
Frontal_Mid_Orb_R	0.18	-0.08	-0.0168	0.0382	-0.262	
Putamen_L	0.81	-0.08			-0.375	0.065
Calcarine_R			-0.805	-0.0535		0.122
Paracentral_Lobule_R			-0.4414	-0.2976		
Cerebellum_8_L			-0.4121			
Lingual_R			-0.3924		-0.292	
Rectus_R			-0.366		-0.081	
Temporal_Pole_Sup_R			-0.3209	0.2698		
Cerebellum_Crus2_R			-0.2809			
Thalamus_R			-0.1715	0.0304		
Postcentral_L		0.23	-0.1314			0.071
Cuneus_R			-0.1037		0.566	
Frontal_Inf_Oper_R			-0.052			0.139
Supp_Motor_Area_R			-0.0422		-0.145	
Cerebellum_Crus1_L			-0.0297	-0.0707		
Cerebellum_6_R			0.0264			
Temporal_Pole_Sup_L			0.038	-0.0213	-0.128	-0.427
Cuneus_L			0.046			
Frontal_Inf_Tri_L			0.0494			
Precentral_R		-0.08	0.1018			
Temporal_Sup_L			0.5282			
Cerebellum_7b_L			0.5321			
Calcarine_L			0.7595			-0.134
Putamen_R					-0.449	0.01
Vermis_6					-0.374	
Cerebellum_6_L					-0.278	
Paracentral_Lobule_L					-0.176	
Temporal_Mid_R					-0.106	-0.153
Caudate_R					0.054	0.01
ParaHippocampal_R					0.092	0.015
Cerebellum_8_R				-0.0562	0.115	-0.028
Frontal_Inf_Orb_L		0.3			0.142	0.214
SupraMarginal_R					0.206	

Occipital_Sup_L			0.29	
Frontal_Sup_Medial_R	-0.59	0.0653	0.303	
Cerebellum_4_5_L		0.7197	0.34	
Rolandic_Oper_L	-0.23	0.0205		0.542
Fusiform_L	-0.11			
Precentral_L	-0.09			
Cingulum_Ant_L	-0.03			
Parietal_Inf_R	-0.02			
Temporal_Mid_L	0.07			0.091
Thalamus_L	0.08	-0.2715		
Supp_Motor_Area_L	0.12			
Vermis_3		-0.7653		
Olfactory_R		-0.4417		
Temporal_Pole_Mid_R		-0.3901		
Cingulum_Ant_R		-0.0409		
Frontal_Sup_Medial_L		-0.0385		
Occipital_Sup_R		-0.0219		
Cerebellum_Crus1_R		0.0134		0.086
Cerebellum_Crus2_L		0.0672		
Cerebellum_7b_R		0.1074		
Vermis_9		0.6321		
ParaHippocampal_L		0.9032		
Occipital_Inf_L				-0.209
Frontal_Med_Orb_R				-0.178
Pallidum_L				-0.172
Pallidum_R				-0.062
Cerebellum_9_L				0.095
Temporal_Sup_R				0.252

Table S2. Abbreviations, terminology, and descriptions for various terms used throughout the paper. Definition is based on common knowledge or referenced in the manuscript.

Abbreviations	Full Text	Description
CFS	Chronic Fatigue Syndrome	Debilitating disorder characterized by chronic widespread pain, fatigue, sleep abnormalities, and cognitive impairment that are worsened by mild to moderate exertion (post-exertional malaise or exertional exhaustion.
GWI	Gulf War Illness	Debilitating disorder characterized by chronic widespread pain, fatigue, sleep abnormalities, and cognitive impairment that are worsened by mild

		to moderate exertion (post-exertional malaise or exertional exhaustion. Experienced by 25 - 30% of veterans of the 1990 - 1991 Gulf War Conflict.
KNN	K Nearest Neighbors algorithm	Characterizes all available classes and classifies new cases according to a similarity measure.
SVM	Linear SVM algorithm	Supervised learning algorithm that seeks to fit a plane to a series of data points.
DT	Decision Tree	Classification algorithm that functions like an "if-then" or flowchart type modeling tool where each node represents a respective classification of the data.
RFE	Random Forest	Random forest is multiple stacks of decision trees, or flowchart like nodes, to classify data with high accuracy.
Adaboost	AdaBoost algorithm	Boosting algorithm that combines multiple weak learners to create a stronger one.
Naïve Bayes	Naive Bayes Classifier	Probabilistic classifier that assumes dependence of the predictor variables on prior data.
QDA	Quadratic Discriminant Analysis algorithm	Quadratic classifier that is a variant of LDA to allow for non linear seperation of data.
Logistic	Logistic Regression	A regression algorithm that fits a line to data according to the logistic function, or logit function where each coefficient represents the log odds.
NN	Neural Net	A classification tool capable of back propagation and self-updating of priors that uses multiple nodes and layers to create an accurate predictive capability.
Ensemble Model	Ensemble Model	A form of predictive algorithm or algorithms that typically uses multiple algorithms to generate a prediction.
Bucket of Models	Bucket of Models	An ensemble learning technique where multiple algorithms are used to select the best overall algorithm for a problem.
Stacking	Stacking	Aggregate multiple weak models to obtain a better prediction
Boosting	Boosting	Sequential training of weak learners to transition them to strong learners
RFE	Recursive Feature Elimination	Adds and removes new features until it determines the best local optimized model at that point in time.
Training Set	Training, Model Build	Dataset used to build, or train, the model
Testing Set	Test, Validation, Hold-out-Sample	Dataset used to validate, or test, the model. Separate from the training sample. Does not overlap.
AAL	Automated Anatomical Labeling Atlas	A standardized brain atlas that seeks to align voxel data with respective coordinates in the MNI space.
MNI	Montreal Neurological Institute	A standardized space developed by the MNI institute that maps voxel data to relevant coordinates on the brain.
Voxel	Voxel	A three dimensional volume that is representative of a unit in an fMRI image. It is sized according to the parameters set by the user (2 mm x 2 mm x 2mm, etc.)

BOLD	Blood-oxygen-level-dependent imaging	Method used in fMRI to visualize areas of blood oxygenation, or activation, in the brain.
fMRI	Functional magnetic resonance imaging	An imaging method that measures brain activity by detecting changes in blood flow.
MPRAGE	Magnetization Prepared Rapid Acquisition Gradient Echo	A form of 3D structural brain imaging that allows one to visualize whole brain coverage in a short period of time.
TR/TE	Repetition Time/ Echo Time	Repetition time is the time between sequential pulses, where as time to echo or echo time is the time between the RF pulse and receipt of the echo.
FoV	Field-of-View	The field the image is fitted to.
EPI	Echo-planar imaging	Form of MRI imaging where multiple echoes of different phase steps are acquired.
FWHM	Full Width at Half Maximum	Form of measurement of a wave form that seeks to find the difference between the two extreme values of a variable equal to half its maximum value.

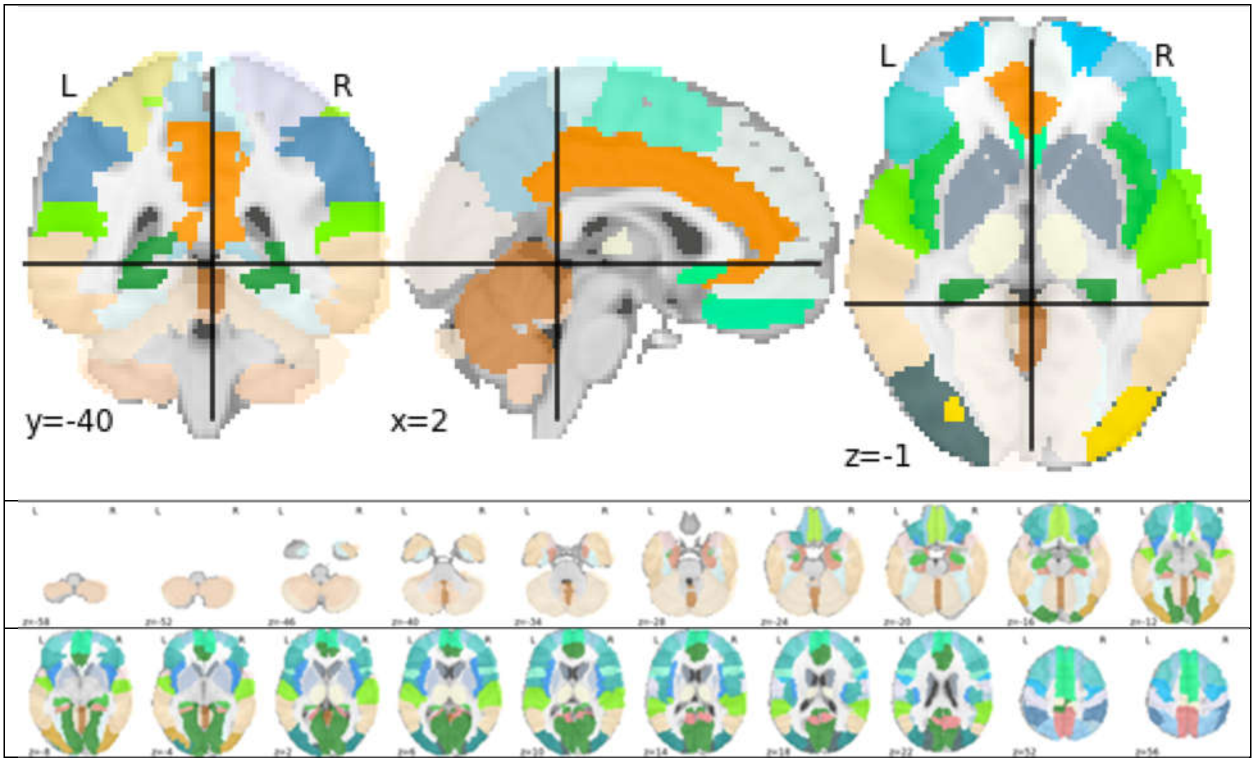

Figure S1. AAL Atlas

Table S3: Atlas centers of mass (MNI coordinates) and voxels per region

Index	L R	Abbreviation	X	Y	Z	Voxels
1	L	Precentral_L	−39	−6	51	3526

2	R	Precentral_R	41	−8	52	3381
3	L	Frontal_Sup_L	−18	35	42	3599
4	R	Frontal_Sup_R	22	31	44	4056
5	L	Frontal_Sup_Orb_L	−17	47	−13	963
6	R	Frontal_Sup_Orb_R	18	48	−14	997
7	L	Frontal_Mid_L	−33	33	35	4863
8	R	Frontal_Mid_R	38	33	34	5104
9	L	Frontal_Mid_Orb_L	−31	50	−10	888
10	R	Frontal_Mid_Orb_R	33	53	−11	1015
11	L	Frontal_Inf_Oper_L	−48	13	19	1038
12	R	Frontal_Inf_Oper_R	50	15	21	1399
13	L	Frontal_Inf_Tri_L	−46	30	14	2529
14	R	Frontal_Inf_Tri_R	50	30	14	2151
15	L	Frontal_Inf_Orb_L	−36	31	−12	1690
16	R	Frontal_Inf_Orb_R	41	32	−12	1707
17	L	Rolandic_Oper_L	−47	−8	14	990
18	R	Rolandic_Oper_R	53	−6	15	1331
19	L	Supp_Motor_Area_L	−5	5	61	2147
20	R	Supp_Motor_Area_R	9	0	62	2371
21	L	Olfactory_L	−8	15	−11	280
22	R	Olfactory_R	10	16	−11	289
23	L	Frontal_Sup_Medial_L	−5	49	31	2992
24	R	Frontal_Sup_Medial_R	9	51	30	2134
25	L	Frontal_Med_Orb_L	−5	54	−7	719
26	R	Frontal_Med_Orb_R	8	52	−7	856

27	L	Rectus_L	−5	37	−18	852
28	R	Rectus_R	8	36	−18	745
29	L	Insula_L	−35	7	3	1858
30	R	Insula_R	39	6	2	1770
31	L	Cingulum_Ant_L	−4	35	14	1400
32	R	Cingulum_Ant_R	8	37	16	1313
33	L	Cingulum_Mid_L	−5	−15	42	1941
34	R	Cingulum_Mid_R	8	−9	40	2203
35	L	Cingulum_Post_L	−5	−43	25	463
36	R	Cingulum_Post_R	7	−42	22	335
37	L	Hippocampus_L	−25	−21	−10	932
38	R	Hippocampus_R	29	−20	−10	946
39	L	ParaHippocampal_L	−21	−16	−21	978
40	R	ParaHippocampal_R	25	−15	−20	1132
41	L	Amygdala_L	−23	−1	−17	220
42	R	Amygdala_R	27	1	−18	248
43	L	Calcarine_L	−7	−79	6	2258
44	R	Calcarine_R	16	−73	9	1861
45	L	Cuneus_L	−6	−80	27	1526
46	R	Cuneus_R	14	−79	28	1424
47	L	Lingual_L	−15	−68	−5	2095
48	R	Lingual_R	16	−67	−4	2300
49	L	Occipital_Sup_L	−17	−84	28	1366
50	R	Occipital_Sup_R	24	−81	31	1413
51	L	Occipital_Mid_L	−32	−81	16	3270

52	R	Occipital_Mid_R	37	−80	19	2098
53	L	Occipital_Inf_L	−36	−78	−8	941
54	R	Occipital_Inf_R	38	−82	−8	989
55	L	Fusiform_L	−31	−40	−20	2310
56	R	Fusiform_R	34	−39	−20	2518
57	L	Postcentral_L	−42	−23	49	3892
58	R	Postcentral_R	41	−25	53	3823
59	L	Parietal_Sup_L	−23	−60	59	2065
60	R	Parietal_Sup_R	26	−59	62	2222
61	L	Parietal_Inf_L	−43	−46	47	2447
62	R	Parietal_Inf_R	46	−46	50	1345
63	L	SupraMarginal_L	−56	−34	30	1256
64	R	SupraMarginal_R	58	−32	34	1974
65	L	Angular_L	−44	−61	36	1173
66	R	Angular_R	46	−60	39	1752
67	L	Precuneus_L	−7	−56	48	3528
68	R	Precuneus_R	10	−56	44	3265
69	L	Paracentral_Lobule_L	−8	−25	70	1349
70	R	Paracentral_Lobule_R	7	−32	68	836
71	L	Caudate_L	−11	11	9	962
72	R	Caudate_R	15	12	9	994
73	L	Putamen_L	−24	4	2	1009
74	R	Putamen_R	28	5	2	1064
75	L	Pallidum_L	−18	0	0	293
76	R	Pallidum_R	21	0	0	280

77	L	Thalamus_L	−11	−18	8	1100
78	R	Thalamus_R	13	−18	8	1057
79	L	Heschl_L	−42	−19	10	225
80	R	Heschl_R	46	−17	10	249
81	L	Temporal_Sup_L	−53	−21	7	2296
82	R	Temporal_Sup_R	58	−22	7	3141
83	L	Temporal_Pole_Sup_L	−40	15	−20	1285
84	R	Temporal_Pole_Sup_R	48	15	−17	1338
85	L	Temporal_Mid_L	−56	−34	−2	4942
86	R	Temporal_Mid_R	57	−37	−1	4409
87	L	Temporal_Pole_Mid_L	−36	15	−34	755
88	R	Temporal_Pole_Mid_R	44	15	−32	1187
89	L	Temporal_Inf_L	−50	−28	−23	3200
90	R	Temporal_Inf_R	54	−31	−22	3557
91	L	Cerebellum_Crus1_L	−35	−67	−29	2603
92	R	Cerebellum_Crus1_R	38	−67	−30	2648
93	L	Cerebellum_Crus2_L	−28	−73	−38	1894
94	R	Cerebellum_Crus2_R	33	−69	−40	2117
95	L	Cerebellum_3_L	−8	−37	−19	136
96	R	Cerebellum_3_R	13	−34	−19	207
97	L	Cerebellum_4_5_L	−14	−43	−17	1125
98	R	Cerebellum_4_5_R	18	−43	−18	861
99	L	Cerebellum_6_L	−22	−59	−22	1694
100	R	Cerebellum_6_R	26	−58	−24	1795
101	L	Cerebellum_7b_L	−31	−60	−45	585

102	R	Cerebellum_7b_R	34	−63	−48	534
103	L	Cerebellum_8_L	−25	−55	−48	1887
104	R	Cerebellum_8_R	26	−56	−49	2308
105	L	Cerebellum_9_L	−10	−49	−46	869
106	R	Cerebellum_9_R	10	−49	−46	809
107	L	Cerebellum_10_L	−22	−34	−42	144
108	R	Cerebellum_10_R	27	−34	−41	159
109	midline	Vermis_1_2	2	−39	−20	53
110	midline	Vermis_3	2	−40	−11	228
111	midline	Vermis_4_5	2	−52	−6	665
112	midline	Vermis_6	2	−67	−15	371
113	midline	Vermis_7	2	−72	−25	194
114	midline	Vermis_8	2	−64	−34	243
115	midline	Vermis_9	2	−55	−35	174
116	midline	Vermis_10	1	−46	−32	112

Table S4. Demographics. Demographic and questionnaire items were significantly different between groups by ANOVA followed by Bonferroni correction for multiple comparisons ($p<0.05$): * CFS vs. control and GWI; ** CFS vs. GWI; *** control vs. CFS and GWI; § all 3 groups significantly different from each other; GWI vs. control and CFS (mean ± SD).

	**Control**	**CFS**	**GWI**
N	30	38	75
Age	45.9 ± 7.4	47.7 ± 13.0	47.8 ± 10.8
Male	63.3%	29.0% *	78.7%
Body mass index	28.5 ± 4.5	26.2 ± 5.4 **	29.4 ± 5.5
**CFS Symptom Severity Questionnaire**			
fatigue	1.3 ± 1.2 ***	3.4 ± 0.8	3.5 ± 0.7
memory	1.2 ± 1.2 ***	2.9 ± 0.9	3.1 ± 0.8
sore throat	0.3 ± 0.7 ***	1.0 ± 1.0	1.4 ± 1.2
lymph nodes	0.1 ± 0.4	1.0 ± 1.1 §	1.5 ± 1.3
muscle pain	0.6 ± 1.0	2.5 ± 1.3 §	3.2 ± 0.9
joint pain	0.8 ± 1.0	1.8 ± 1.5 §	3.2 ± 1.0
headache	1.0 ± 1.2	2.0 ± 1.3 §	2.7 ± 1.2
sleep	1.7 ± 1.4 ***	3.2 ± 0.9	3.5 ± 0.8

exertional exhaustion	0.6 ± 1.1 ***	3.5 ± 0.8	3.4 ± 0.9
**SF-36 Quality of Life**			
physical functioning	84.7 ± 24.4 ***	45.9 ± 26.6	45.6 ± 23.2
role physical	79.3 ± 37.2 ***	9.5 ± 25.2	7.9 ± 22.4
bodily pain	82.3 ± 20.2	46.6 ± 27.0 §	28.1 ± 17.5
general health	68.9 ± 22.6 ***	34.7 ± 23.7	25.8 ± 18.5
vitality	59.8 ± 21.0 ***	18.9 ± 15.9	16.8 ± 15.0
social functioning	79.3 ± 25.3 ***	32.4 ± 27.4	29.8 ± 22.7
role emotional	86.2 ± 31.5	69.4 ± 44.7	29.2 ± 38.1
mental health	73.9 ± 17.0	67.7 ± 17.0	53.9 ± 22.1

Table S5. Sensitivity, Specificity, False Discovery Rate (FDR), PPV, and NPV for the highest performing overall model. Although individually selected regions may have performed higher on different regions, this model produced the best overall accuracy across all models and one of the highest accuracies (82% for Logistic Regression and Decision Tree) on the validation set overall

	Nearest Neighbors	Linear SVM	Decision Tree	Random Forest	AdaBoost	Naive Bayes	QDA	Logistic Regression	Neural Net
				Day 1					
Sensitivity	24%	55%	66%	39%	47%	68%	45%	55%	55%
Specificity	93%	89%	90%	99%	96%	58%	100%	86%	86%
FDR	40%	30%	24%	6%	14%	57%	0%	34%	34%
PPV	60%	70%	76%	60%	60%	60%	60%	60%	60%
NPV	72%	81%	85%	72%	72%	72%	72%	72%	72%
				Day 2					
Sensitivity	34%	58%	63%	37%	53%	39%	45%	58%	58%
Specificity	89%	86%	86%	95%	90%	89%	100%	86%	86%
FDR	41%	33%	31%	22%	29%	38%	0%	33%	33%
PPV	59%	67%	69%	78%	71%	63%	100%	67%	67%
NPV	74%	81%	83%	76%	80%	75%	79%	81%	81%
.									
